# Supplementary material for: Machine Learning Technology Reveals the Concealed Interactions of Phytohormones on Medicinal Plant In Vitro Organogenesis
Source: Biomolecules. 2020 May 11;10(5):746. doi: 10.3390/biom10050746 (PMC7278175; doi:10.3390/biom10050746)
Supplement: Supplementary file 1 [file biomolecules-10-00746-s001.pdf]

Supplementary Material

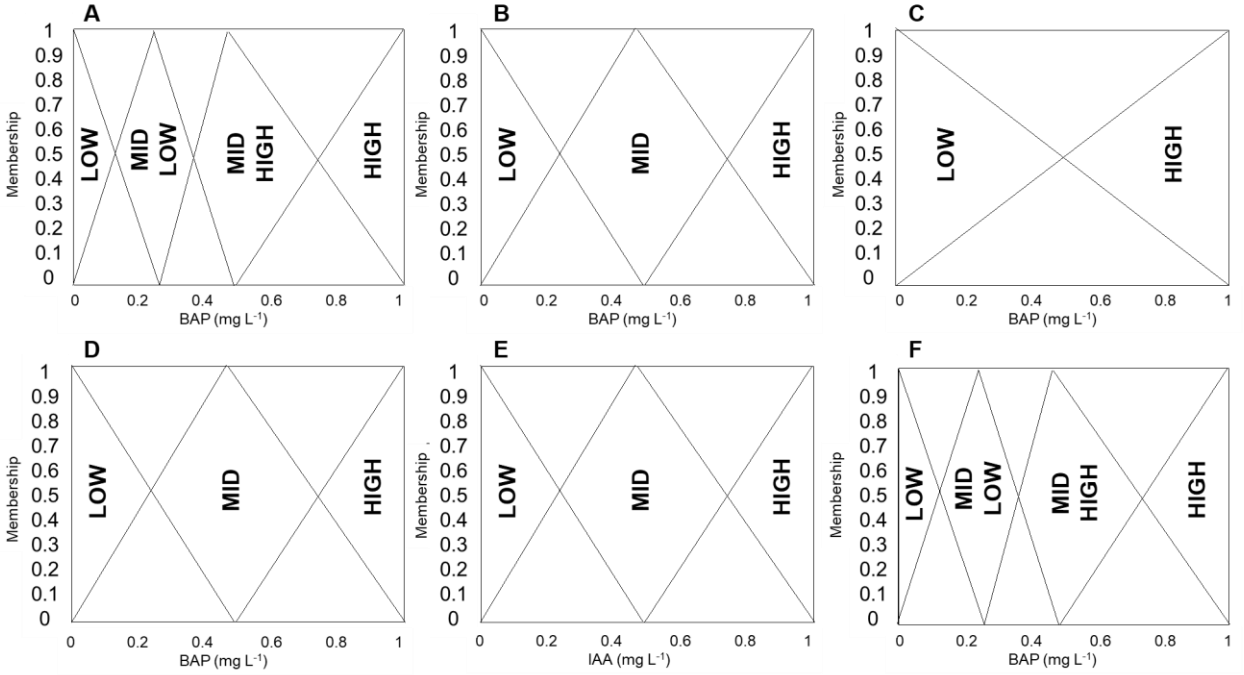

Figure S1. Graphical interpretation of phytohormone concentration ranging provided by FormRules® for each output: (**A**) BAP concentrations for %DS; (**B**) BAP concentration for %IS; (**C**) BAP concentration for NDS; (**D**) BAP concentration for NIS; (**E**) IAA concentration for NIS; (**F**) BAP concentration for %CAL.
